# Editorial: Novel technologies for enrichment, extraction, and determination of phenolic compounds in foods, volume I

**DOI:** 10.3389/fnut.2023.1238748

**Published:** 2023-06-27

**Authors:** Yu Xiao, Baoru Yang, Runqiang Yang

**Affiliations:** ^1^College of Food Science and Technology, Hunan Agricultural University, Changsha, China; ^2^Department of Biochemistry and Food Chemistry, University of Turku, Turku, Finland; ^3^College of Food Science and Technology, Whole Grain Food Engineering Research Center, Nanjing Agricultural University, Nanjing, Jiangsu, China

**Keywords:** phenolic compounds, enrichment, extraction, determination, food

Phenolic compounds, a class of plant secondary metabolites with high biological activity, are mainly composed of flavonoids, phenolic acids, and anthocyanins (1). Due to the large amount of active phenolic hydroxyl groups in phenolics, they have a strong ability to scavenge free radicals and have various physiological and biochemical functions, including anti-cancer, anti-tumor, anti-aging, and other health-promoting functions (2). However, they cannot be synthesized in the human body and need to be ingested through external sources (3). Therefore, the novel technologies for enrichment, extraction, and determination of phenolic compounds have received widespread attention.

Phenolic compounds are widely distributed in vegetables, fruits and grains (4). The metabolomics approaches based on mass spectrometry can achieve comprehensive extraction and determination of different phenolic compounds. Zhang et al. found that isoflavones, sinapic acid derivatives, catechin and epicatechin, phenolic alcohols, chlorogenic acid, and lignans were the main phenolic compounds in soybean, rapeseed, peanut skin, olive, sunflower seed, sesame and flaxseed, respectively. Liu H.-Y. et al. reported that four phenolic classes with 316 phenolic metabolites were identified in white radish. Liu S. et al. concluded that *Citrus* L. fruit, as a functional fruit, was also rich in health-promoting phytonutrients and bioactive compounds, such as flavonoids, phenolic acids, vitamins, carotenoids, pectins, and fatty acids. A thorough understanding of phenolic components among different species may provide scientific guidance for better enrichment and extraction of specific phenolic compounds and benefit the extension of the value chain of functional foods.

In recent years, it has become a major development trend in enhancing the activity of phenolic compounds through biotic or abiotic stress (5, 6), which is of great significance for the extension of the food industry chain. Villamil-Galindo et al. showed that Ellagitannins and UVA radiation were proved to be efficient in biofortify strawberry agro-industrial by-products, significantly improving the phenolic compounds content and their bioactive properties with adequate bioaccessibility, adding value to the strawberry agro-industrial by-products. Wang M. et al. found that UV-B radiation promoted the accumulation and synthesis of isoflavones in soybean hypocotyls and cotyledon callus tissues, particularly malonyl isoflavones. Also, a combination of ultrasound and exogenous GABA treatment can be used to produce mung bean sprouts with enriched polyphenols content and enhanced antioxidant activity (Wang L. et al.). Xie et al. investigated that ultrasound-assisted aqueous two-phase (ATP) extraction was used as an effective method to achieve the simultaneous separation and preliminary purifification of phenolics from grape pomace. Interestingly, Hong et al. found that ultrasonic washing was an abiotic elicitor to induce the accumulation of phenolics in fruit and vegetables while retaining quality attributes and microbial safety. Besides, fermentation is an effective way to increase the content of endogenous phenolic compounds in plants. Yang J. et al. found that the content of total organic acids, phenols and flavonoids increased in fermented ginkgo biloba kernel juice by *Lactobacillus plantarum* Y2, which effectively improved the nutritional value and safety of ginkgo biloba kernel juice. Wang K. et al. showed that the nutritional value and bioactivity of black beans were enhanced when fermented as tempeh, and the total respective levels of phenolics, flavonoids, and proanthocyanidins released from black bean tempeh were 1.21, 1.40, and 1.55 times higher than those of unfermented black beans following *in vitro* digestion, respectively. Zheng et al. comprehensively investigated three modification methods (shear emulsifying, ball milling, and autoclave treatment) to improve the structure, physicochemical properties, phenolic compounds, and antioxidant capacity of food.

These studies emphasize phenolic-rich foods have become a major trend in the future. The technologies for enrichment, extraction, and determination of phenolic compounds in foods are becoming increasingly abundant, which has an important contribution to improving food nutrition and processing quality, and developing healthy foods.

In general, the article discussed in this editorial analyzes the composition of phenolic compounds in different plants, explores new technologies for enriching and extracting phenolic compounds, and demonstrates the potential of enhancing the activity of phenolic compounds and increasing their production to extend the food industry chain. As our understanding of phenolic compounds and their novel technologies continues to grow, it can provide valuable supplements to existing technologies for enrichment, extraction, and determination of phenolic compounds, and contribute to improving the nutritional value and industrial added value of food.

The articles discussed in this editorial demonstrate the potential of phenolic-rich foods as a promising dietary intervention in fighting against obesity. It would be more and more clear that they may offer a valuable addition to existing weight loss and metabolic health interventions and ultimately contribute to the development of more effective and holistic approaches to obesity prevention and treatment.

## Author contributions

YX: writing—original draft and writing—review and editing. BY: writing—review and editing. RY: investigation and writing—review and editing. All authors contributed to the article and approved the submitted version.
